# The UK Breast Cancer in Pregnancy (UKBCiP) Study. Incidence, diagnosis, management and short-term outcomes of breast cancer first diagnosed during pregnancy in the United Kingdom: A population-based descriptive study

**DOI:** 10.3310/nihropenres.13652.2

**Published:** 2025-01-06

**Authors:** Claudia Hardy, Andrew Brand, Julie Jones, Marian Knight, Philip Banfield

**Affiliations:** 1 Obstetrics and Gynaecology, Glan Clwyd Hospital, Bodelwyddan, LL18 5UJ, UK; 2 North Wales Organisation for Randomised Trials in Health & Social Care (NWORTH), College of Medicine and Health, Bangor University, Bangor, Gwynedd, LL57 2PZ, UK; 3 Glan Clwyd Hospital, Bodelwyddan, LL18 5UJ, UK; 4 National Perinatal Epidemiology Unit, Nuffield Department of Population Health, University of Oxford, Oxford, OX3 7LF, UK

**Keywords:** Breast cancer, pregnancy, incidence

## Abstract

**Background:**

The incidence of breast cancer first arising during pregnancy has been estimated in several countries to be 2.4-7.8/100,000 births, but has not been established in the United Kingdom (UK). We aimed to estimate the incidence of breast cancer diagnosed during pregnancy in the UK and to describe its management and short-term outcomes for mothers and babies.

**Methods:**

This population-based descriptive study used the UK Obstetric Surveillance System (UKOSS). Cases were prospectively identified through monthly UKOSS mailings to all UK consultant-led maternity units. All cases of breast cancer diagnosed first during pregnancy, between October 1, 2015, and September 30, 2017, were eligible, with 84 confirmed cases analysed. Women with breast cancer diagnosed before pregnancy or with a recurrence were excluded. The primary outcomes were the incidence of breast cancer first diagnosed during pregnancy, maternal mortality, severe maternal morbidity, perinatal mortality, and severe neonatal morbidity.

**Results:**

The incidence was 5.4/100,000 maternities (95% CI 4.37, 6.70). Nine women (11%) had undergone *in vitro* fertilisation (IVF), compared with a contemporaneously estimated 2.6% IVF pregnancies in the UK. During pregnancy, 30 women (36%) underwent surgery and 37 (44%) received chemotherapy. Three women had major maternal morbidity during pregnancy. Two women died and two perinatal deaths occurred.

**Conclusions:**

The incidence of breast cancer arising in pregnancy in the UK is similar to that reported elsewhere. The higher proportion of IVF pregnancies among affected women needs further investigation, as it may not be entirely explained by relatively advanced maternal age. With caveats, management followed that outside pregnancy, but there was considerable variation in practice. Although short-term outcomes were generally good for mothers and babies, a larger prospective study is required. Iatrogenic pre-term delivery and its associated risks to the infant can often be avoided; treatment was administered during pregnancy without evidence of harms to the infant.

## Plain Language Summary

Breast cancer is the most common cancer in the UK. We wanted to know how common it is for women in the UK to be diagnosed with breast cancer for the first time while pregnant, and what clinical treatment these women received.

Every month, all maternity units in the UK send anonymous information about pregnant women who have certain rare conditions diagnosed to a central database, the UK Obstetric Surveillance System (UKOSS). We looked at all the information that UKOSS collected during a two-year period about women who were newly diagnosed with breast cancer during pregnancy.

During the two years, 84 pregnant women were diagnosed with breast cancer for the first time. Based on this, we estimated that there were 5.4 new diagnoses of breast cancer per 100,000 women giving birth in the UK. This is similar to the numbers that researchers have estimated in other countries. The women received broadly similar clinical treatment to non-pregnant women with breast cancer. However, treatment varied a lot between individuals.

More diagnosed women than would be expected had undergone IVF. This finding might be partly related to the older age of the pregnant women diagnosed with breast cancer. However, this finding needs further investigation.

It is easy to dismiss breast cancer symptoms during pregnancy as normal pregnancy breast changes. This can delay diagnosis. Although breast cancer during pregnancy is rare, women should discuss any symptoms with their healthcare providers. Midwives should be alert to the possibility of breast cancer and consider investigating symptoms further.

## Introduction

Breast cancer is the most common malignancy in the United Kingdom (UK), accounting for almost one-third (30%) of cancers in women in England ^1^, Wales ( https://phw.nhs.wales/services-and-teams/welsh-cancer-intelligence-and-surveillance-unit-wcisu/cancer-reporting-tool-official-statistics/cancer-incidence/), Scotland ^2^, and Northern Ireland ( http://www.qub.ac.uk/research-centres/nicr/).

The incidence of breast cancer rises with age. The fact that many women are delaying pregnancy until later in life may lead to an increasing incidence of breast cancer arising for the first time in pregnancy. Births to women aged 40 years or older increased by 9.5% between 2006–07 and 2017–18 in England ^3^. Studies elsewhere have identified an increasing trend in the incidence of breast cancer during pregnancy; for example, in Sweden between 1963 and 2002 ^4^.

Breast Cancer Associated with Pregnancy (BCAP) is defined as breast cancer diagnosed during pregnancy or lactation up to 12 months postpartum, but some studies on this subject include cases diagnosed up to 5 years after delivery. Breast cancer arising during and after delivery appear to be two separate entities with different behaviors and outcomes ^5– 9^. Thus, the estimated incidence of BCAP ranges from 1/3,000 to 1/10,000 pregnancies, depending on the study population and definition used ^4, 10– 12^. It is estimated that 18%–33% of BACP cases are diagnosed specifically during pregnancy ^4, 10– 12^. Based on this observation, the incidence of breast cancer diagnosed for the first time during pregnancy can be estimated in other countries to range from 2.4 to 7.8 cases per 100,000 births ^4, 10– 12^, but the incidence has not been previously estimated in the UK.

The aim of this study was to identify cases of breast cancer diagnosed during pregnancy, a period when diagnosis, staging, and treatment can be challenging for women, their families, and clinicians. The objective was that these data could inform clinical discussions with women and their families in order to co-produce management decisions that must account for optimal maternal therapy as well as fetal well-being, in what is a rare situation for both clinicians and patients.

## Methods

### Patient and public involvement

No patients were directly involved in the design of this study. The public was involved in the design of the study as part of the UKOSS Steering Committee.

All newly diagnosed cases of breast cancer during pregnancy in the UK were reported through the UK Obstetric Surveillance System (UKOSS) in women who delivered their babies or had a termination of pregnancy or miscarriage between October 1, 2015, and September 30, 2017. The total number of maternities in the UK over the 2 years of the study was used as the denominator to calculate the incidence. The incidence was calculated with 95% confidence intervals.

Women whose breast cancer had been diagnosed before pregnancy or who had recurrence of previously diagnosed disease were excluded.

UKOSS collects information on specific rare events that occur during pregnancy, agreed on by a national steering committee, from all obstetric units in the UK. The reporters submit monthly returns for the current list of conditions, including zero values, notifying UKOSS when an event has occurred (in this case, the diagnosis of breast cancer in pregnancy). UKOSS staff sent the data collection form ( https://www.npeu.ox.ac.uk/assets/downloads/ukoss/forms/UKOSS-Breast-Cancer-V1.pdf) for completion when notified of a case. In addition, we notified oncologists around the UK of the study to gain their assistance with the completion of information on oncological diagnosis and management. The UKOSS methodology ensures that no patient identifiable data are included for analysis while excluding duplicate submissions ^13^. Cases were allocated UKOSS identification numbers, and all data were anonymised. Once received, duplicate cases were excluded by comparison of key clinical information. Reminders were sent if forms were not returned or were incomplete.

Ethics committee approval was obtained (Wales Research Ethics Committee 5, January 15, 2015, EC 14/WA/1267, IRAS project ID 165517). Consent was not required for the collection of anonymous routine data. Betsi Cadwaladwr University Health Board sponsored and funded this study.

The information requested included maternal demographics, details of the current and past pregnancies, BRCA (BReast CAncer) gene mutation status, and details of the diagnosis and staging when known. Information about staging, surgical management, chemotherapy use, and pregnancy outcomes was also collected. Babies were categorised as small for gestational age (SGA) if the birth centile was less than the 10 ^th^ centile ^14^.

Categorical data are summarised as percentages. Continuous data are presented as medians, IQR and ranges. Group comparisons were performed using Fisher’s exact test.

We used the STROBE cross sectional reporting guidelines ^15^.

## Results

A total of 132 cases were reported during the 24-month follow-up period. Of these, 29 cases were excluded as they did not meet the eligibility criteria, and 8 cases were duplicate notifications identified by UKOSS. The data collection form was not returned for 11 cases; therefore, 84 confirmed cases were available for analysis.

### Incidence

The estimated total maternities in the UK during the study period were 1,544,700 ^16, 17^ ( https://www.opendata.nhs.scot/dataset/births-in-scottish-hospitals), giving an estimated incidence of 5.4 per 100,000 maternities (95% CI [4.37, 6.70]).

### Maternal characteristics

The characteristics of the women are shown in Table 1. The median maternal age at diagnosis was 36 years (IQR: 33–38 years; range: 26–44 years). The median BMI at booking was 25 (IQR: 22–28.5; range: 18–48). Nine women (11%) had in vitro fertilisation (IVF) pregnancies.

**Table 1.** Characteristics of women.

Characteristic	Median (IQR *, range) or No. of women (%)
Age at diagnosis	36 (33 – 38, 26 – 44) N = 84 (100%)
26 – 30	14 (17%)
31 – 35	30 (36%)
36 – 40	33 (39%)
41 – 45	6 (7%)
Missing	1 (1%)
Body mass index (BMI) (kg/m ^2^)	25 (22 – 28.5, 18 – 48) N = 84 (100%)
<20	5 (6%)
20 – 25	44 (52%)
26 – 30	22 (26%)
31 – 35	8 (10%)
36 – 40	4 (5%)
>40	1 (1%)
IVF pregnancy?	
Yes	9 (11%)
No	75 (89%)
Known to have BRCA mutation?	
Yes	3 (4%)
No	69 (82%)
Missing	12 (14%)

*IQR = interquartile range.

Data on presentation and diagnosis are presented in Table 2. Two women were asymptomatic; their tumours were identified at specific screening arising from known familial increased risk. The majority of symptomatic women (n=50, 61%) presented solely with a lump. Fourteen women (17%) presented with a lump associated with other breast symptoms, including pain, skin changes, discharge, or tenderness. The remaining 18 women (22%) presented with different combinations of symptoms but not a lump, including nipple inversion, pain and fullness, skin changes, breast enlargement, discharge from the nipple, and erythema. The median duration of symptoms before diagnosis was three weeks (IQR, 2–9 weeks; range, 0–56 weeks) and the median gestational age at diagnosis was 25 weeks (IQR: 16–33 weeks; range: 2–40 weeks) ( Table 2).

**Table 2.** Gestational age, symptoms and size of tumour at diagnosis.

Characteristic	Median (IQR *, range) or No. of patients (%)
Gestational age at diagnosis (weeks)	25 (16 – 33, 2 – 40) N = 84 (100%)
<14	18 (21%)
14 – 27	23 (28%)
28 – 36	32 (38%)
37 – > 36	5 (6%)
Missing	6 (7%)
Symptoms	
Asymptomatic	2 (2%)
Symptomatic	76 (91%)
Missing	6 (7%)
Lump only	50 (60%)
Lump with or without other symptoms	70 (83%)
Pain / tenderness with or without other symptoms	13 (16%)
Skin changes / nipple inversion with or without other symptoms	10 (12%)
Other symptoms	2 (2%)
Clinical size of tumour at diagnosis (mm)	26 (20 – 40, 7 – 120) N = 84 (100%)
≤ 20	14 (17%)
20 – 50	37 (44%)
> 50	6 (7%)
Missing	27 (32%)
Interval from symptoms to diagnosis (weeks)	3 (2 – 9, 0 – 56) N = 84 (100%)
0 – 4	45 (53%)
5 – 9	10 (12%)
10 – 14	8 (9%)
15 – 19	1 (1%)
20 – 24	1 (1%)
>24	5 (6%)
Missing	10 (18%)

*IQR = interquartile range.

### Staging and tumour characteristics

Staging investigations performed during pregnancy were reported fully for 76 women. Staging involved multiple combinations of modalities and included breast ultrasound (n = 73), mammogram (n = 62), chest X-ray (n = 30), liver ultrasound (n = 28), chest and/or abdominal computed tomography (n = 24), breast MRI (n = 21), echocardiogram (n = 15), bone scan (including radionuclear) (n = 13), non-contrast abdominal, skeletal or brain MRI (n = 10), and PET (n = 3). Twenty-four women received at least breast ultrasound, chest X-ray, and mammogram.

Data are available on the histology type in 71 women. The histological type was invasive ductal carcinoma in 62 (74%) patients, poorly differentiated in 2 (2%), invasive lobular carcinoma in 3 (4%), and ductal carcinoma in situ (DCIS) in 2 (2%). Metaplastic, inflammatory, and invasive ductal and lobular carcinomas were reported in the remaining cases.

Of the 61 cases in which the grade was recorded, grade 3 tumours were found in 45 (74%), grade 2 in 15 (25%), and grade 1 in 1 case (1%).

The TNM (tumour size, lymph nodes, metastasis) stage was known in 58 women ( Table 3). The clinical tumour size at diagnosis was greater than 2 cm in 39 patients (68%). Just over one in six women (N=9) had a tumour greater than 5 cm. Using the American Joint Committee on Cancer (AJCC) pathological staging system, 14 (24%) were stage 1, 27 (46%) were stage 2, 13 (22%) were stage 3, and 4 (7%) were stage 4. Of the 58 women with complete TNM staging, 24 (41%) were node-negative, 34 (59%) were node-positive, and 4 (7%) had distant metastasis to the liver and bones.

**Table 3.** Tumour characteristics and staging in relation to tumour receptor findings.

Characteristic	ER (+) 41 (49%)	ER (-) 29 (35%)	Total * 84 (100%)
Histological grade			
1	0 (0%)	0 (0%)	1 (1%)
2	12 (29%)	3 (10%)	15 (18%)
3	25 (60%)	19 (66%)	45 (53%)
N/A	1 (2%)	2 (7%)	4 (5%)
Missing	4 (9%)	5 (17%)	19 (23%)
Histological type			
Ductal	37 (90%)	23 (79%)	62 (74%)
Lobular	1 (2%)	1 (3%)	3 (4%)
Ductal and lobular	1 (2%)	0 (0%)	1 (1%)
Metaplastic	0 (0%)	1 (3%)	1 (1%)
Poorly differentiated	0 (0%)	2 (7%)	2 (2%)
DCIS	2 (5%)	0 (0%)	2 (2%)
Missing	0 (0%)	2 (7%)	13 (16%)
Distribution of cancer			
Localised	26 (63%)	22 (76%)	51 (61%)
Multifocal	11 (27%)	4 (14%)	15 (18%)
Missing	4 (10%)	3 (10%)	18 (21%)
PR status			
Positive	23 (56%)	1 (3%)	25 (30%)
Negative	5 (12%)	26 (90%)	31 (37%)
Missing	13 (32%)	2 (7%)	28 (33%)
HER2 Status			
Positive	15 (37%)	8 (28%)	24 (28%)
Negative	25 (61%)	20 (69%)	45 (54%)
Missing	1 (2%)	1 (3%)	15 (18%)
Pathological T stage			
T1	14 (34%)	6 (21%)	20 (24%)
T2	17 (42%)	11 (38%)	30 (36%)
T3	5 (12%)	6 (21%)	12 (14%)
T4	1 (2%)	2 (7%)	4 (5%)
Missing	4 (10%)	4 (13%)	18 (21%)
N Stage			
N0	14 (34%)	10 (35%)	25 (30%)
N1	10 (25%)	5 (17%)	17 (21%)
N2	3 (7%)	4 (14%)	7 (8%)
N3	3 (7%)	4 (14%)	7 (8%)
Missing	11 (27%)	6 (20%)	28 (33%)
M Stage			
M0	39 (95%)	26 (90%)	68 (81%)
M1	1 (2%)	2 (7%)	4 (5%)
Missing	1 (2%)	1 (3%)	12 (14%)

ER, oestrogen receptor; HER2, human epidermal growth factor receptor 2; PR, progesterone receptor; DCIS, ductal carcinoma in situ *ER status unknown for 14 women.

Tumour receptor status in relation to other tumour characteristics and staging is shown in Table 3. Overall, 41 (59%) patients were ER (+), 24 (35%) were Her2 (+) and 20 (29%) were triple-negative (ER, PR, HER2). Tumour characteristics and staging in relation to hormonal receptors are shown in Table 3. Of the nine women who conceived by IVF, four had ER (+) tumours, four had ER (-) tumours, and ER status information was missing for one.

### Treatment

The frequencies of surgery and chemotherapy by trimester of diagnosis are presented in Table 4. Data on the use of systemic chemotherapy were available for 79 women. This was administered during pregnancy in 37 cases (47%), was not recommended in 7 cases (9%), and was delayed until the end of pregnancy in 35 cases (44%). The timing was unknown in 5 cases. Of the 37 women who received chemotherapy during pregnancy, 18 (49%) received neoadjuvant chemotherapy and 17 (46%) received adjuvant chemotherapy. One woman received palliative chemotherapy for metastatic disease.

**Table 4.** Treatment approaches by trimester of diagnosis.

	Surgery during pregnancy				Chemotherapy during pregnancy			
Gestational age at diagnosis	Yes	Not indicated	Delayed until end of pregnancy	Not known	Yes	Not indicated	Delayed until end of pregnancy	Not known
**<14 weeks** **(n = 18)**	13	3	1	1	11	5	2	0
**14–27 weeks** **(n = 23)**	11	4	8	0	20	0	3	0
**28–36 weeks** **(n = 32)**	6	6	20	0	5	2	25	0
**>36 weeks** **(n = 5)**	0	0	5	0	0	0	5	0
**Not known** **(n = 6)**	0	0	0	6	1	0	0	5

The median gestational age at the start of chemotherapy was 23 weeks (IQR, 19–27.5; range, 13–35 weeks).

Of the 18 women diagnosed during the first trimester, 11 received chemotherapy during pregnancy (all after 14 weeks), two received chemotherapy after miscarriage and in five cases chemotherapy was not indicated. Of these 18 women, 13 underwent surgery during pregnancy, all between 6 and 15 weeks.

Of the 23 women diagnosed during the second trimester, 20 had chemotherapy during pregnancy and 3 had the start of chemotherapy delayed until after delivery. In all three cases, delivery occurred after 37 completed weeks. In the same group of women, 11 had surgery during pregnancy and 8 had it delayed until the end of pregnancy (in two of these cases, the delivery was between 34 and 36 weeks); all other cases delivered after 37 weeks.

Breast cancer was diagnosed in 37 women during their third trimester. Thirty-five of them had induction of labour or pre-labour caesarean section, in 50% of the cases before 37 completed weeks (between 32 and 36 weeks). Of these women diagnosed during the third trimester, 6 underwent surgery during pregnancy and 25 had delayed surgery until the end of pregnancy. Five patients received chemotherapy during pregnancy, and 30 had it delayed until the end of the pregnancy.

Overall, thirty women out of the 77 with data (39%) underwent surgery during pregnancy: 14 underwent breast conservation surgery, and 16 underwent mastectomy. One woman underwent both procedures during pregnancy. The median gestational age at breast conservation surgery was 17 weeks (range: 6–34 weeks). The median gestational age at mastectomy was 18 weeks (range: 9–35 weeks). Surgery was delayed until the end of pregnancy in 34 (44 %) women. Of the 34 women, 11 received neoadjuvant chemotherapy during pregnancy. The reasons for the delayed surgery were not collected.

Including the 32 cases known that had axillary clearance during pregnancy, the median number of lymph nodes removed was 12 (IQR: 4.8–17.3 range 2 to 34). In these cases, the median number of positive nodes was 1 (IQR: 0–3.3 range: 0–16).

Chemotherapy regimens used during pregnancy and outcomes are given in Table 5. The systemic regimen that most patients received was anthracycline-based chemotherapy (31 of 37). This was associated with cyclophosphamide use in 26 of the 37 women who received chemotherapy during pregnancy. Taxanes were used in 15 women. The most widely used combination was FEC (fluorouracil, epirubicin, and cyclophosphamide) in ten women. Five women received trastuzumab during pregnancy, including in combination with pertuzumab. These women generally had advanced disease, evidenced by one or more of large primary tumour, multiple affected lymph nodes, or metastatic disease. No congenital abnormalities, NICU admissions or stillbirths were reported in the infants of any of the four who received trastuzumab during pregnancy and did not subsequently terminate the pregnancy. No other HER2-targeted therapy was received during pregnancy. A further woman who received doxorubicin and cyclophosphamide during pregnancy gave birth at 28 weeks to start Herceptin (trastuzumab) postnatally.

**Table 5.** Chemotherapy regimens used during pregnancy and outcomes.

Chemotherapy	Number of cases	GA start (median *)	GA end (median *)	GA delivery (median *)	Outcome: mother	Outcome: neonate
FEC	10	24	33 (not known in 6)	38	No complications	1 NICU: prematurity 34 weeks, 2 cases SGA
EC	6	24.5	28.5 (not known in 4)	36	1 urinary infection	3 NICU: 1 prematurity, 1 jaundice, 1 GBS infection. 1 case SGA
FEC-T	5	16	32 (not known in 2)	36	1 pericarditis postnatal	1 SROM 33 weeks, 2 NICU admissions: prematurity, 1 case SGA
EC-D	3	26	33 (not known in 2)	38	Extravasation, burn left arm, 1 woman metastatic disease	1 NICU: chest infection, 1 case SGA
Docetaxel	2	23	33 (not known in 1)	37	No complications	1 case SGA
Paclitaxel and Trastuzumab	2	32	Not known	40	1 case metastasis, pleural effusion	1 case SGA 1 case TOP
AC	2	22.5	Not known	32	No complications	1 delivered at 28 weeks to start Herceptin. Died of sepsis
6 Other combinations ** . 1 case unknown	7 Each one in one case	13 – 35	26 – 36	28 – 40	5 cases no complications. 2 cases worsened condition in mother	1 stillbirth 1 admission to NICU for prematurity 1 SGA Other 4 no complications

*Gestational ages in the “other” category are presented as ranges because the patients received different treatment regimens. **Three of these women received trastuzumab in combination with other chemotherapy, including in one case with pertuzumab. In nine cases, chemotherapy was continued postpartum, but the gestational age at which it was stopped antenatally is unknown. FEC: fluorouracil, epirubicin, and cyclophosphamide. EC: epirubicin and cyclophosphamide. FEC-T: fluorouracil, epirubicin, cyclophosphamide, and docetaxel. EC-D: epirubicin, cyclophosphamide, and docetaxel. AC: doxorubicin and cyclophosphamide. GA: gestational age. NICU: neonatal intensive care unit. SGA: small for gestational age, includes cases with a birth weight below the 10 ^th^ centile. GBS: group B *Streptococcus*. SROM: spontaneous rupture of membranes. TOP: termination of pregnancy.

### Outcomes

There were 81 babies born to the 80 women who continued with pregnancy, with a median gestational age at delivery of 37 completed weeks (IQR: 35–38, range 28–41). In total, 41% of the babies were delivered preterm and 59% after at ≥37 completed weeks. Outcomes in neonates born preterm and at ≥37 completed weeks are summarised in Table 6. All 16 women who gave birth before 36 completed weeks received steroids for fetal lung maturation; two babies died, both prior to 30 weeks. Sixteen babies (20%) were admitted to the neonatal care unit; 13 of these (81%) were admitted because of symptoms directly related to their prematurity. At birth, 11 of 78 (14%) neonates with known birth weight were classified as small for gestational age (SGA; weight below the 10 ^th^ centile) ^14^. Eight of the babies who were SGA were born to the 35 mothers who had received chemotherapy, and three SGA babies were born to the 39 mothers who did not receive chemotherapy during pregnancy. This difference was not statistically significant (22.8% vs. 7.7%, P = 0.142; Fisher’s exact test).

**Table 6.** Outcomes in neonates born preterm and at ≥37 completed weeks.

Outcome	Preterm infants (<37 weeks’ gestation at birth) (n = 33)	Infants born at ≥37 completed weeks (n = 48)
Neonatal death	2	0
NICU admission	14	2

Denominator is infant (n=81). There was one multiple birth.

Thirty women were induced, 28 (93%) for reasons related to their cancer. The mode of birth was known for 78 of the 80 women who continued pregnancy beyond the second trimester; 37 (47.4%) had pre-labour caesarean section, 21 (57%) after 37 weeks and 16 (43%) preterm. Of the 41 women who were planning a vaginal birth, 27 (66%) had a spontaneous vaginal birth, four (10%) had a ventouse birth, three (7%) had a forceps birth, one woman had a breech birth and six women (15%) had an emergency caesarean section after the onset of labour.

Three women were diagnosed later in pregnancy with metastatic cancer in the liver, thoracic spine, and lung, respectively. Two maternal deaths were reported. Two additional women had major maternal morbidity during pregnancy or puerperium; the morbidity in one of these cases was thought to be directly related to chemotherapy.

Thirty-seven percent of women (27 out of 73 known) breastfed, and 19 had lactation suppression.

## Discussion

### Main findings

The incidence of breast cancer diagnosed during pregnancy in the UK (5.4 per 100,000 maternities) is similar to the figures of 2.4 to 7.8 cases per 100,000 births estimated in the USA, Sweden, and Western Australia ^4, 10– 12^.

The median maternal age was 36 years, which is the same as that found in the Prospective Study of Outcomes in Sporadic versus Hereditary Breast Cancer (POSH Breast Cancer Study) in the UK between 2000 and 2008, which included 2956 young women aged 18–40 years with breast cancer ^18^, with a similar median ^19^ and mean ^20– 23^ maternal age found in other studies during pregnancy: 35–37. In contrast, during the study period, the maternal age was less than 35 years in 78.5% of births in the UK ^24^. This may partially explain the disproportionate number of women with IVF pregnancies who tend to be older. However, the percentage of IVF pregnancies in the UK in 2015 and 2016 was 2.57% and 2.6% ^25^, respectively, compared to 11% in this study. This observation requires further investigation. While there is uncertainty, there is a need to discuss this potential additional risk, and special attention should be paid to breast symptoms and examinations during the antenatal period in women who conceive by IVF until the position is clarified.

Throughout the period of the study, pregnant women in the UK were overweight or obese in more than 47% of cases ^26^. We found a median BMI of 25 in women presenting with breast cancer during pregnancy, with 42% of cases above the normal BMI. This finding is similar to the median found in the POSH study ^18^. This tendency may reflect previously reported findings of lower BMI among premenopausal women with breast cancer ^27^.

The high percentage of symptomatic women before diagnosis (97%) is similar to that found in the POSH study (98%) ^18^ and in the CARING study (93%), a retrospective study of cancer diagnosed in pregnancy at 14 UK sites between 2016 and 2020 ^19^. This high percentage is as might be expected outside a screening program population’s demographic. In postmenopausal women, two-thirds are asymptomatic at the time of diagnosis, with diagnosis at an earlier stage of screening. This may contribute to the worse prognosis in young women with breast cancer ^28^. Three-quarters of the cases presented with a breast lump, two-thirds on its own and the remainder mostly with a lump and/or other symptoms. These other presentations, such as nipple inversion, pain and fullness, skin changes, breast enlargement, nipple discharge, and erythema, need to be highlighted to women and midwives because symptoms are easily dismissed as part of the normal pregnancy breast changes. This emphasises the importance of increasing awareness of this diagnosis when discussing breast symptoms at any stage of pregnancy.

Pregnancy often masks symptoms and signs of breast cancer, leading to delayed diagnosis. The interval between the start of symptoms and diagnosis reported here (median 3 weeks; IQR: 2–9) is shorter than the median time of 4 weeks (range 1–104 weeks) found in the Australian study of Ives ^10^, which included postpartum diagnosis, and the mean time of 3.9 months during pregnancy found by Langer *et al.* ^29^.

The median gestational age at diagnosis, 25 weeks, had a wide range, similar to the findings of other, smaller studies ^22^. A large number of women (44%) were diagnosed in the third trimester. Of these 37 women, 35 (95%) underwent induction of labour or pre-labour caesarean section. In most cases, treatment (surgery or chemotherapy) was delayed until the end of pregnancy with iatrogenic delivery between 32 and 36 weeks in nearly half (49%). This finding could reflect guidelines regarding safe delivery time for women receiving chemotherapy to allow both maternal and fetal bone marrow recovery ^30, 31^. The effects of granulocyte-colony stimulating factor on treating neutropenia in pregnancy may modify this decision, avoiding iatrogenic prematurity, and seems to be safe ^32, 33^.

The UK confidential inquiry into maternal mortality, Mothers and Babies: Reducing Risk through Audit and Confidential Enquiries (MBRRACE-UK), has recently reported on a subset of the cases identified through the UKOSS reporting system as part of this UK breast cancer in pregnancy (UKBCiP) study, with direct access to anonymised patient records for assessment. This suggests new guidance: in general, early delivery to avoid delays in chemotherapy should not be recommended ^34^. For women diagnosed with breast cancer in the third trimester, the risk–benefit is likely to favour both mother and baby if a woman can receive at least two cycles of chemotherapy prior to a term (39–40 weeks) birth ^30^.

Overall, we found that women were more likely to be induced (57% vs. 39%) or undergo elective caesarean section (47% vs. 15%) than the general population in England ^32^. This is similar to the 42% caesarean section rate in women with breast cancer observed in the CARING study ^19^.

The Prospective Study of Outcomes in Sporadic versus Hereditary Breast Cancer (POSH Breast Cancer Study) ^18^, although not directly comparable, provides context outside pregnancy because the demographics overlap with this UKBCiP study. In the POSH study, breast cancer was found to present at a more advanced stage in pregnancy, with higher node involvement and tumour grade. The tumour size at diagnosis was more commonly greater (> 2 cm in 68% vs. 52% of cases and > 5 cm in 15% vs. 7% in pregnancy and outside pregnancy, respectively). Node involvement was greater (59% vs. 50%), as were metastases (7% vs. 2.5%). The histological grade may be higher in pregnancy (grade 1, 1% vs. 6%; grade 2, 18% vs. 32.9%; and grade 3, 53% vs. 59%). The finding of invasive ductal carcinoma in 84% of cases is similar to that reported by other authors ^10, 21– 23, 29, 35^. Despite 2/3 (68%) being diagnosed with stage II or III disease, most women did well in the short-term. In our study, four women (5%) presented with stage IV disease. Notably, staging was not conducted or was incomplete for around a third of women. At the time of the surveillance, UK guidelines on breast cancer in pregnancy recommended staging for metastases only in cases with high clinical suspicion ^36^; however, the 2019 MBRRACE report subsequently found multiple examples of cases in which a lack of staging information impaired decision-making around management, including resulting in unnecessarily invasive surgery, and concluded that guidance is needed to ensure timely staging ^37^.

The finding that 59% of women with newly diagnosed breast cancer in pregnancy were oestrogen receptor (ER) positive compares to 66% in the POSH study, with triple-negative tumours present in 29% and 20% of women, respectively.

Although in England, overall for the period 2016–17 7.9% of babies were preterm ^38^ and in Scotland 6.5% ( https://www.opendata.nhs.scot/dataset/births-in-scottish-hospitals), the preterm birth rate of 41% was lower than that observed in the CARING study (47%) ^19^ and that reported by Amant *et al.* in an international collaborative study of 311 cases of breast cancer diagnosed during pregnancy (49.6%) ^6^. A smaller case series reported higher rates of preterm delivery; Gomez-Hidalgo in 11 cases found that 54.6% of the neonates were preterm ^20^ and Framarino-dei-Malatesta in 54.5% of 22 women ^22^. Outcome data suggest that the fetus does relatively well, even when exposed to several maternal chemotherapy regimens. Amant *et al.* (2015) concluded that prenatal exposure to maternal cancer, with or without treatment, did not impair the cognitive, cardiac, or general development of children in early childhood. Prematurity was correlated with worse cognitive outcomes; however, this effect was independent of cancer treatment ^39^.

The safety of current chemotherapy regimens during pregnancy is well documented, and breast cancer treatment during pregnancy should adhere to that of non-pregnant women. Most patients (31 of 37) received anthracycline-based chemotherapy. This was associated with cyclophosphamide use in 26 of the 37 women who received chemotherapy during pregnancy. Taxanes were used in 15 women. The most widely used combination was FEC in ten women.

It seems that later delivery (for the fetus), providing appropriate chemotherapy in pregnancy, and aiming for vaginal delivery are all reasonably safe. In a situation that is psychologically distressing for a woman and her family, the creation of ‘normality’ in relation to the birthing experience can be hugely important to their well-being and future perception.

### Strengths and limitations of this study

This is the first national prospective study of breast cancer diagnosed during pregnancy in the UK and its incidence, management, and short-term outcomes. The study raised awareness of the rare condition of breast cancer in pregnancy among UK obstetricians and oncologists during the 2 years of the UKOSS data collection.

UKOSS data rely on the reporting of monthly cases, and a certain amount of under-reporting may occur; hence, the use of a 95% confidence interval has been included with our estimate of incidence.

The incidence estimate reported here must be considered a minimum estimate for several reasons. UKOSS is a system that involves all consultant-led maternity units in the UK, but some women diagnosed with breast cancer during pregnancy could have chosen to undergo termination of pregnancy or had a miscarriage before reaching these maternity units; thus, this will not be a true reflection of what may be happening in early pregnancy.

Additionally, although we wrote to clinical oncology units and contacted patient support groups such as Mummy’s Star to raise awareness of the study, the anonymised nature of UKOSS reporting precludes the study team from having women’s details, for example, through self-reporting, to cross-check with reporting units specifically to ensure their details had been included in the study. Currently, pregnancy data are not included in most cancer registries; therefore, these data cannot be used to enhance case ascertainment.

## Conclusions

The incidence of breast cancer arising during pregnancy in the UK is 5.4 per 100,000 maternities. The relatively high number of IVF pregnancies requires further investigation as this may not be related to increased age alone. This study confirms relatively late presentation, with diagnosis at a more advanced stage, highlighting the need for education of women and those who care for them regarding breast symptoms in pregnancy. Breast symptoms should be discussed specifically, and women should be encouraged to report any concerns with a low threshold for seeking investigation or additional medical review.

With the exceptions that chemotherapy should not be administered in the first trimester and that the use of HER-2 targeted therapies should generally be avoided, the management of these women should be the same as in non-pregnant women. Standard chemotherapy can be safely delivered during pregnancy with good overall fetal outcomes. Iatrogenic pre-term delivery and its associated risks to the infant can therefore often be avoided.

Co-ordinated multidisciplinary work among obstetricians, breast surgeons, and oncologists is essential to ensure optimal management.

A larger prospective study is required to allow longer-term follow-up, but this would require individual patient consent. Meanwhile, adding pregnancy status to the UK cancer registries would yield more information about treatment and outcomes.

## Data Availability

Data cannot be shared because of confidentiality issues and the potential identifiability of sensitive data, as identified by the Research Ethics Committee approval. Requests to access the data can be made by contacting the National Perinatal Epidemiology Unit data access committee via general@npeu.ox.ac.uk. The estimated response time for requests is 4 weeks. Data sharing outside the UK or European Union may require consultation with the UK Health Research Authority. For more information, please refer to the National Perinatal Epidemiology Unit Data Sharing Policy available at https://www.npeu.ox.ac.uk/assets/downloads/npeu/policies/Data_Sharing_Policy.pdf
