# Supplementary material for: The UK Breast Cancer in Pregnancy (UKBCiP) Study. Incidence, diagnosis, management and short-term outcomes of breast cancer first diagnosed during pregnancy in the United Kingdom: A population-based descriptive study
Source: NIHR Open Res. 2025 Jan 6;4:40. Originally published 2024 Jul 15. [Version 2] doi: 10.3310/nihropenres.13652.2 (PMC11372348; doi:10.3310/nihropenres.13652.2)
Supplement: Peer review history

# Reviewer report: Reviewer response for version 2

**DOI:** 10.3310/nihropenres.15028.r34366

**Published:** 2025-01-20

**Authors:** Nimmi Kapoor

**Affiliations:** 1 University of California Los Angeles, Los Angeles, California, USA

No further comments.

Is the work clearly and accurately presented and does it cite the current literature?

Partly

If applicable, is the statistical analysis and its interpretation appropriate?

Partly

Are all the source data underlying the results available to ensure full reproducibility?

No source data required

Is the study design appropriate and is the work technically sound?

Partly

Are the conclusions drawn adequately supported by the results?

Partly

Are sufficient details of methods and analysis provided to allow replication by others?

Partly

Reviewer Expertise:

Clinical outcomes in breast cancer, breast ultrasound, pregnancy and breast cancer, benign disease of the breast, surgical education

I confirm that I have read this submission and believe that I have an appropriate level of expertise to confirm that it is of an acceptable scientific standard.

# Reviewer report: Reviewer response for version 1

**DOI:** 10.3310/nihropenres.14823.r32620

**Published:** 2024-09-06

**Authors:** Nimmi Kapoor

**Affiliations:** 1 University of California Los Angeles, Los Angeles, California, USA

This paper utilizes an incomplete set of data from 7-9 years ago to attempt to describe the incidence of breast cancer diagnosed during pregnancy in the UK. Understanding breast cancer in pregnancy is very important, with rising maternal age and increased incidence of breast cancer in young women, but this manuscript falls short in many areas.

 Major criticism #1: The primary objective set by the authors are not met accurately. The authors provide a calculation of incidence of 5.4 per 100,000 maternities. This number is flawed and not explained. The numerator includes women who terminated pregnancies, and they exclude 11 patients without information which is 11.6% of the data. The denominator is termed “maternities”, yet it will miss the large number of women who were pregnant and did not carry the pregnancy to delivery. Some of these excluded women would have been included in the numerator.

  Major criticism #2: The authors are using a dataset from 2015-2017 for no clear reason, with missing stage information for over 30% of patients.  This is not of great relevance.

 Minor critiques are below:

 Abstract

 This statement is unnecessary: “With caveats, the management followed that outside pregnancy, but there was considerable variation in practice.” 

 This is not clear or relevant: “It is often possible to avoid exposing the baby to iatrogenic prematurity.”

 Methods

 See major comment #1

 This is inaccurate: “No patients were involved in this study.”

 Results

 See major comment #2

 Organization of chemotherapy, surgery, and trimester timing of treatment is unclear.  Consider a table sorted by trimester, tumor stage, receipt of chemotherapy, type of surgery, and then maternal and fetal outcomes.

 Combine Tables 1 and 2, consider removing clinical stage from the table since it is missing for so many women (could either delete or simply report in text). You don’t need to report all the data in Table 2 in text format for gestational age.

 Please clarify what is meant by, “Staging investigations performed during pregnancy were reported fully for 76 women and included various modalities and combinations” i.e. bone scan, CXR, MRI, ultrasound, etc. If possible, provide numbers and combinations of these modalities.  

 Clarify, “delayed until the end of pregnancy.” Does this mean after pregnancy/childbirth, in the postpartum period?  This term, “the end of pregnancy” is used several times throughout, please consider re-wording.

 Table 4. 2 patients received trastuzumab during pregnancy?  This is generally contraindicated.  Can you comment and/or correct.  In the legend for this table, you list “AC-TH and ECX” but do not have this in the table anywhere. Please list abbreviations in order of appearance from left column down then to the right.

 Discussion:

 If the primary outcome is incidence and maternal/neonatal short term outcome, more discussion should be around these items rather than on stage and tumor type for which this study is lacking far too much information.

 Consider emphasizing number of women presenting with Stage IV disease. 

 Please comment on the CARING study that was recently published regarding diagnosis and outcome of cancer diagnosed during pregnancy in the UK: Br J Cancer. 2024 May;130(8):1261-1268.

Is the work clearly and accurately presented and does it cite the current literature?

Partly

If applicable, is the statistical analysis and its interpretation appropriate?

Partly

Are all the source data underlying the results available to ensure full reproducibility?

No source data required

Is the study design appropriate and is the work technically sound?

Partly

Are the conclusions drawn adequately supported by the results?

Partly

Are sufficient details of methods and analysis provided to allow replication by others?

Partly

Reviewer Expertise:

Clinical outcomes in breast cancer, breast ultrasound, pregnancy and breast cancer, benign disease of the breast, surgical education

I confirm that I have read this submission and believe that I have an appropriate level of expertise to state that I do not consider it to be of an acceptable scientific standard, for reasons outlined above.

## References

[1] : CAnceR IN PreGnancy (CARING) - a retrospective study of cancer diagnosed during pregnancy in the United Kingdom. *Br J Cancer* .2024;130(8) : 10.1038/s41416-024-02605-x 1261-1268 10.1038/s41416-024-02605-x 38383704 PMC11014900

# Response

**Published:** 2024-11-26

**Authors:** Ruth Tunn

**Affiliations:** NPEU, University of Oxford, Oxford, England, UK

*Thank you for your constructive comments. The authors have written the responses below, which I am submitting on their behalf. Ruth Tunn, Research Facilitator, National Perinatal Epidemiology Unit, University of Oxford.*

***Reviewer Comments:***

*1. Major criticism #1: The primary objective set by the authors are not met accurately. The authors provide a calculation of incidence of 5.4 per 100,000 maternities. This number is flawed and not explained. The numerator includes women who terminated pregnancies, and they exclude 11 patients without information which is 11.6% of the data. The denominator is termed “maternities”, yet it will miss the large number of women who were pregnant and did not carry the pregnancy to delivery. Some of these excluded women would have been included in the numerator.*

**Author Response: **The use of maternities as the denominator when calculating incidence is the standard approach in population-based studies of incidence in perinatal epidemiology. This is an approximation and will, as you indicate, miss women who do not carry the pregnancy to birth. However, it is necessary to use this metric as it is impossible to capture every early pregnancy loss and termination in a national population. Using maternities as the denominator is a standardised approach, making incidence estimates comparable between countries.

 Excluding from the case group women who terminated pregnancies would be counterproductive as it is important not to lose potential learning from these cases; in addition, the condition being examined may be a causal factor in the decision to terminate a pregnancy that might otherwise have continued.

 ---------------

***Reviewer Comments:***

*2. Major criticism #2: The authors are using a dataset from 2015-2017 for no clear reason, with missing stage information for over 30% of patients.  This is not of great relevance.*

**Author Response: **The UK Obstetric Surveillance System (UKOSS) conducts a rolling programme of prospective surveillance of specified uncommon conditions occurring during pregnancy. Surveillance of breast cancer was conducted between 2015 and 2017. Full staging information is unfortunately not available for around a third of patients because comprehensive staging investigations were not universally conducted during pregnancy. This in itself is an important finding. At the time of the surveillance, UK guidelines on breast cancer in pregnancy ( https://www.rcog.org.uk/media/2q4jb0kz/gtg_12.pdf) recommended staging for metastases only in cases with high clinical suspicion; however, the 2019 MBRRACE report ( https://www.npeu.ox.ac.uk/assets/downloads/mbrrace-uk/reports/MBRRACE-UK%20Maternal%20Report%202019%20-%20WEB%20VERSION.pdf) subsequently found multiple examples of cases in which a lack of staging information impaired decision-making around management, and concluded that guidance is needed to ensure timely staging.

 We have added this information to the Discussion section of the manuscript.

 Although some time has passed since the data were collected, this remains the only population-based study to estimate incidence of breast cancer during pregnancy in the UK. To provide benchmark information and to avoid research waste, we feel it is important to make these data publicly available.

 ---------------

***Reviewer Comments:***

*Minor critiques are below:*

*Abstract*

*3. This statement is unnecessary: “With caveats, the management followed that outside pregnancy, but there was considerable variation in practice.”*

**Author Response: **We have opted to retain this sentence because one of our stated objectives was to describe case management. Although we do not have space in the abstract to describe the varied management in detail, we wish to signpost that the article provides further information.

 ---------------

***Reviewer Comments:***

*4. This is not clear or relevant: “It is often possible to avoid exposing the baby to iatrogenic prematurity.”*

**Author Response: **We have rephrased this sentence as follows:

 “Iatrogenic pre-term delivery and its associated risks to the infant can often be avoided; treatment was administered during pregnancy without evidence of harms to the infant”.

 We have also clarified a corresponding sentence in the conclusion.

 ---------------

***Reviewer Comments:***

*Methods*

*See major comment #1*

*5. This is inaccurate: “No patients were involved in this study.”*

**Author Response: **This was intended to refer to patient and public involvement in study design. We have clarified the statement as follows:

 “No patients were directly involved in the design of the study”.

 ---------------

***Reviewer Comments:***

*Results*

*See major comment #2*

*6. Organization of chemotherapy, surgery, and trimester timing of treatment is unclear.  Consider a table sorted by trimester, tumor stage, receipt of chemotherapy, type of surgery, and then maternal and fetal outcomes.*

**Author Response: **We have added the following sentence and a new table (Table 4) to the manuscript:

 "The frequencies of treatment approaches by trimester of diagnosis are presented in Table 4."

 Given the relatively small number of cases, stratifying the data risks the possibility of deductive disclosure. We have therefore limited the number of stratifying variables.

 ---------------

***Reviewer Comments:***

*7. Combine Tables 1 and 2, consider removing clinical stage from the table since it is missing for so many women (could either delete or simply report in text). You don’t need to report all the data in Table 2 in text format for gestational age.*

**Author Response: **We have opted to keep Tables 1 and 2 separate for clarity, and have retained clinical stage in the table for ease of access/extraction. We have retained the mention of median gestational age in the text but have deleted the additional details that duplicated the table content unnecessarily.

 ---------------

***Reviewer Comments:***

*8. Please clarify what is meant by, “Staging investigations performed during pregnancy were reported fully for 76 women and included various modalities and combinations” i.e. bone scan, CXR, MRI, ultrasound, etc. If possible, provide numbers and combinations of these modalities.*

**Author Response: **Staging involved multiple combinations of modalities and included breast ultrasound (n = 73), mammogram (n = 62), chest X-ray (n = 30), liver ultrasound (n = 28), chest and/or abdominal computed tomography (n = 24), breast MRI (n = 21), echocardiogram (n = 15), bone scan (including radionuclear) (n = 13), non-contrast abdominal, skeletal or brain MRI (n = 10), and PET (n = 3). Twenty-four women received at least breast ultrasound, chest X-ray, and mammogram.

 We have added this information to the Results.

 ---------------

***Reviewer Comments:***

*9. Clarify, “delayed until the end of pregnancy.” Does this mean after pregnancy/childbirth, in the postpartum period?  This term, “the end of pregnancy” is used several times throughout, please consider re-wording.*

**Author Response: **This refers to the end of pregnancy by whatever means it ended – this could be birth, stillbirth, miscarriage or termination.

 ---------------

***Reviewer Comments:***

*10. Table 4. 2 patients received trastuzumab during pregnancy?  This is generally contraindicated.  Can you comment and/or correct.  In the legend for this table, you list “AC-TH and ECX” but do not have this in the table anywhere. Please list abbreviations in order of appearance from left column down then to the right.*

**Author Response: **In Table 4 (Table 5 in the revised manuscript), we indicate that trastuzumab (with paclitaxel) was used during pregnancy in 2 cases; in one of these cases, the pregnancy was subsequently terminated and in one the infant was small for gestational age.

 A further three women received trastuzumab during pregnancy in combination with other chemotherapy (included in the “other combinations” in Table 4 [now Table 5]), including in one case with pertuzumab. We have clarified this in a footnote to Table 4 (now Table 5). The women who received HER2-targeted therapy generally had advanced disease, evidenced by one or more of large primary tumour, multiple affected lymph nodes, or metastatic disease.

 No congenital abnormalities, NICU admissions or stillbirths were reported in the infants of any of the four women who received trastuzumab during pregnancy and did not subsequently terminate the pregnancy.

 No other HER2-targeted therapy was received during pregnancy.

 A further woman who received doxorubicin and cyclophosphamide during pregnancy gave birth at 28 weeks to start Herceptin (trastuzumab) postnatally (as indicated in Table 4 [now Table 5]).

 We have added this information to the “Treatment” section of the results. We have revised the order of abbreviations in the footnote and removed irrelevant abbreviations.

 ---------------

***Reviewer Comments:***

*Discussion:*

*11. If the primary outcome is incidence and maternal/neonatal short term outcome, more discussion should be around these items rather than on stage and tumor type for which this study is lacking far too much information.*

**Author Response: **Our primary aim was to describe incidence; we have expanded the discussion of incidence with more specific information about findings from past studies in different countries for comparison. As mentioned in response to major comment #2, the lack of staging information in this study is in itself is an important finding, so we have added a comment on this to the Discussion section in the context of the discussion of staging.

 ---------------

***Reviewer Comments:***

*12. Consider emphasizing number of women presenting with Stage IV disease.*

**Author Response: **We have added a sentence in the Discussion emphasising that four women (5%) presented with metastatic disease. We have also discussed the lack of staging investigations in a relatively high proportion of women, which can impair informed decision-making about treatment.

 ---------------

***Reviewer Comments:***

*13. Please comment on the CARING study that was recently published regarding diagnosis and outcome of cancer diagnosed during pregnancy in the UK: Br J Cancer. 2024 May;130(8):1261-1268.*

**Author Response: **We have referred to the CARING study at points in the discussion where relevant comparable data are provided disaggregated by primary tumour site. It is unclear how comparable maternal outcomes in the CARING study are because follow-up duration is not explicitly specified, but it seems likely that some women were followed up for considerably longer than in our design.

# Reviewer report: Reviewer response for version 1

**DOI:** 10.3310/nihropenres.14823.r32623

**Published:** 2024-09-03

**Authors:** Hatem Azim

**Affiliations:** 1 Tecnologico de Monterrey, San Pedro Garza Garcia, Mexico

A well written and needed report. Below are few points for the authors to consider

 - Provide if HER2 targeted therapy were received during pregnancy.

 - provide difference in neonatal outcomes between those who were delivered at full term versus those before week 37. It is relevant to discuss impact of prematurity on the observed neonatal outcomes.

Is the work clearly and accurately presented and does it cite the current literature?

Yes

If applicable, is the statistical analysis and its interpretation appropriate?

Yes

Are all the source data underlying the results available to ensure full reproducibility?

Yes

Is the study design appropriate and is the work technically sound?

Yes

Are the conclusions drawn adequately supported by the results?

Yes

Are sufficient details of methods and analysis provided to allow replication by others?

Yes

Reviewer Expertise:

Breast cancer in the young and its relation to pregnancy and fertility

I confirm that I have read this submission and believe that I have an appropriate level of expertise to confirm that it is of an acceptable scientific standard.

# Response

**Published:** 2024-11-26

**Authors:** Ruth Tunn

**Affiliations:** NPEU, University of Oxford, Oxford, England, UK

*Thank you for your constructive comments. The authors have written the responses below, which I am submitting on their behalf. Ruth Tunn, Research Facilitator, National Perinatal Epidemiology Unit, University of Oxford.*

*1. Provide if HER2 targeted therapy were received during pregnancy.*

 Thank you for your supportive review. In Table 4 (Table 5 in the revised manuscript), we indicate that trastuzumab with paclitaxel was used during pregnancy in 2 cases; in one of these cases, the pregnancy was subsequently terminated and in one the infant was small for gestational age.

 A further three women received trastuzumab during pregnancy in combination with other chemotherapy (included in the “other combinations” in Table 5), including in one case with pertuzumab. We have clarified this in a footnote to Table 5. The women who received HER2-targeted therapy generally had advanced disease, evidenced by one or more of large primary tumour, multiple affected lymph nodes, or metastatic disease.

 No congenital abnormalities, NICU admissions or stillbirths were reported in the infants of any of the four who received trastuzumab during pregnancy and did not subsequently terminate the pregnancy.

 No other HER2-targeted therapy was received during pregnancy. A further woman who received doxorubicin and cyclophosphamide during pregnancy gave birth at 28 weeks to start Herceptin (trastuzumab) postnatally (as indicated in Table 5).

 We have added this information to the “Treatment” section of the results.

 2. *Provide difference in neonatal outcomes between those who were delivered at full term versus those before week 37. It is relevant to discuss impact of prematurity on the observed neonatal outcomes.*

 Thank you for this suggestion. We have added a new table (Table 6) to the manuscript to present this information.

# Reviewer report: Reviewer response for version 1

**DOI:** 10.3310/nihropenres.14823.r32364

**Published:** 2024-08-06

**Authors:** Qing Ting Tan

**Affiliations:** 1 KK Women’s and Children’s Hospital, Singapore, Singapore

The authors’ effort in producing this article is commendable. Data collection for breast cancer in pregnancy is frequently plagued with difficulties as obstetric data is not usually entered into breast cancer registries. The use of a nation-wide database, like UKOSS, helps to provide consistent and vital obstetric information. 

 The paper is generally well-written, data is presented clearly and conclusions are reasonable. 

 Below are some suggestions for improvement

 1) Data was only collected for two years from 2015 to 2017. Collecting data across more years would add to the study population and allow more robust conclusions to be drawn from the results.

 2) It would be easier to understand the different treatment plans within each trimester if patients were divided into groups eg. neoadjuvant chemotherapy and surgery, surgery and adjuvant chemotherapy, surgery only, palliative chemotherapy, no/declined treatment.

 3) The paragraph describing treatment in the third trimester can go after the paragraph describing treatment in the second trimester to make for an easier read.

 4) The literature thus far does not show conclusive evidence of IVF causing an increase in breast cancer risk. Nonetheless, the observation regarding higher IVF rates in this group of pregnant patients deserves further investigations. In the current paper, it would be useful to know the tumour estrogen receptor status in these patients.

 I support and approve the indexing of this paper with some minor suggestions for clarity.

Is the work clearly and accurately presented and does it cite the current literature?

Yes

If applicable, is the statistical analysis and its interpretation appropriate?

I cannot comment. A qualified statistician is required.

Are all the source data underlying the results available to ensure full reproducibility?

Yes

Is the study design appropriate and is the work technically sound?

Yes

Are the conclusions drawn adequately supported by the results?

Yes

Are sufficient details of methods and analysis provided to allow replication by others?

Yes

Reviewer Expertise:

I am a breast surgeon with research and clinical experience in breast cancer in young women and pregnancy associated breast cancer

I confirm that I have read this submission and believe that I have an appropriate level of expertise to confirm that it is of an acceptable scientific standard.

# Response

**Published:** 2024-11-26

**Authors:** Ruth Tunn

**Affiliations:** NPEU, University of Oxford, Oxford, England, UK

*Thank you for your constructive comments. The authors have written the responses below, which I am submitting on their behalf. Ruth Tunn, Research Facilitator, National Perinatal Epidemiology Unit, University of Oxford.*

*1) Data was only collected for two years from 2015 to 2017. Collecting data across more years would add to the study population and allow more robust conclusions to be drawn from the results.*

 Response: Thank you for your constructive comments. Although we agree that a longer study duration would yield informative data, resources are limited and we aim to maximise the number of conditions in pregnancy that the UK Obstetric Surveillance System (UKOSS) can be used to study. We therefore consider the existing limited incidence data for the condition in question when planning each UKOSS study to estimate the minimum duration of surveillance that will be needed to collect a useful amount of data. This avoids overburdening UKOSS reporters while maximising the value of the UKOSS resource overall.

*2) It would be easier to understand the different treatment plans within each trimester if patients were divided into groups eg. neoadjuvant chemotherapy and surgery, surgery and adjuvant chemotherapy, surgery only, palliative chemotherapy, no/declined treatment.*

 Response: We have added the following sentence and a new table (Table 4) to the manuscript:

*"*The frequencies of surgery and chemotherapy by trimester of diagnosis are presented in Table 4. *"*

*3) The paragraph describing treatment in the third trimester can go after the paragraph describing treatment in the second trimester to make for an easier read.*

 Response: Thank you, we have moved the paragraph as suggested to improve the flow of the results.

*4) The literature thus far does not show conclusive evidence of IVF causing an increase in breast cancer risk. Nonetheless, the observation regarding higher IVF rates in this group of pregnant patients deserves further investigations. In the current paper, it would be useful to know the tumour estrogen receptor status in these patients.*

 Response: As we note, this finding might be partly related to the older age of the pregnant women diagnosed with breast cancer, but we have added information on oestrogen receptor status as suggested in the section “Staging and tumour characteristics”.
